# miR-133a function in the pathogenesis of dedifferentiated liposarcoma

**DOI:** 10.1186/s12935-018-0583-2

**Published:** 2018-06-26

**Authors:** Peter Y. Yu, Gonzalo Lopez, Danielle Braggio, David Koller, Kate Lynn J. Bill, Bethany C. Prudner, Abbie Zewdu, James L. Chen, O. Hans Iwenofu, Dina Lev, Anne M. Strohecker, Joelle M. Fenger, Raphael E. Pollock, Denis C. Guttridge

**Affiliations:** 1 Arthur G. James Comprehensive Cancer Center, The Ohio State University, Columbus, OH USA; 2 College of Medicine, The Ohio State University, Columbus, OH USA; 3 Division of Surgical Oncology, Department of Surgery, The Ohio State University, Columbus, OH USA; 4 Division of Medical Oncology, Department of Internal Medicine, The Ohio State University, Columbus, OH USA; 5 Biomedical Informatics, Internal Medicine in the Division of Medical Oncology, The Ohio State University, Columbus, OH USA; 6 Department of Pathology & Laboratory Services, The Ohio State University, Columbus, OH USA; 7 Department of Surgery, Sheba Medical Center, Tel Aviv, Israel; 8 Department of Cancer Biology and Genetics, The Ohio State University, Columbus, OH USA; 9 Department of Veterinary Clinical Sciences, College of Veterinary Medicine, The Ohio State University, Columbus, OH USA

**Keywords:** Sarcoma, Dedifferentiated liposarcoma, miR-133a, Metabolism

## Abstract

**Background:**

Sarcomas are malignant heterogeneous tumors of mesenchymal derivation. Dedifferentiated liposarcoma (DDLPS) is aggressive with recurrence in 80% and metastasis in 20% of patients. We previously found that miR-133a was significantly underexpressed in liposarcoma tissues. As this miRNA has recently been shown to be a tumor suppressor in many cancers, the objective of this study was to characterize the biological and molecular consequences of miR-133a underexpression in DDLPS.

**Methods:**

Real-time PCR was used to evaluate expression levels of miR-133a in human DDLPS tissue, normal fat tissue, and human DDLPS cell lines. DDLPS cells were stably transduced with miR-133a vector to assess the effects in vitro on proliferation, cell cycle, cell death, migration, and metabolism. A Seahorse Bioanalyzer system was also used to assess metabolism in vivo by measuring glycolysis and oxidative phosphorylation (OXPHOS) in subcutaneous xenograft tumors from immunocompromised mice.

**Results:**

miR-133a expression was significantly decreased in human DDLPS tissue and cell lines. Enforced expression of miR-133a decreased cell proliferation, impacted cell cycle progression kinetics, decreased glycolysis, and increased OXPHOS. There was no significant effect on cell death or migration. Using an in vivo xenograft mouse study, we showed that tumors with increased miR-133a expression had no difference in tumor growth compared to control, but did exhibit an increase in OXPHOS metabolic respiration.

**Conclusions:**

Based on our collective findings, we propose that in DDPLS, loss of miR-133a induces a metabolic shift due to a reduction in oxidative metabolism favoring a Warburg effect in DDLPS tumors, but this regulation on metabolism was not sufficient to affect DDPLS.

**Electronic supplementary material:**

The online version of this article (10.1186/s12935-018-0583-2) contains supplementary material, which is available to authorized users.

## Background

Human liposarcoma is the most common adult soft-tissue sarcoma histologic subtype, and includes well-differentiated/atypical lipomatous tumor (WDLPS) and dedifferentiated liposarcoma (DDLPS) [1]. WDLPS and DDLPS share similar 12q13-15 chromosomal amplification and supernumerary ring or giant chromosomes, but DDLPS exhibits more aggressive biological behavior with frequent multiple recurrences and subsequent metastasis in spite of multimodality treatment approaches [2]. The identification of 12q13-15 chromosomal amplifications and MDM2/CDK4 overexpression in patients with DDLPS has provided insight into the genetic changes underlying the disease. The overall 5-year survival rate is approximately 40–60%. However, no significant improvement in survival times has been achieved in the past 35 years [3]. It is likely that until the molecular alterations that contribute to the development and progression of DDLPS are further identified and characterized, discovering novel targets for therapeutic intervention will remain challenging.

MiRNAs are evolutionarily conserved non-coding strands of RNA ~ 22 nucleotides in length that act as expression modulators of genes involved in fundamental cell processes, and their dysregulation have been implicated in multiple cancers [4, 5]. Many miRNAs have been identified to function as either tumor suppressors or oncogenes [6]. There are emerging data that seem to suggest that miRNAs may have a causal role in sarcomagenesis with potential therapeutic implications. Our understanding of miRNAs in liposarcoma is limited, but several profiling studies led to the identification of candidate tumor suppressor and oncogenic miRNAs. Other studies have proposed that miR-25, miR-26a, miR-92a, miR-145, and miR-155 function as potential therapeutic targets for DDLPS management [7–11]. These data imply miRNAs might act as drivers in the pathogenesis of DDLPS.

To this end, we recently characterized miRNA expression signatures associated with human liposarcoma tissue compared to paired subjacent normal tissue [12]. Unexpectedly, we found that several muscle-specific miRNAs (myomiRs), miR-1, -133a, and -206, were significantly underexpressed in liposarcoma compared to their subjacent normal tissues. These miRNAs play key roles in muscle differentiation and proliferation [13]. However, new findings indicate that myomiRs are involved in other processes such as adipocyte differentiation [14–16], chondrocyte differentiation [17], immunological responses [18], and mitochondrial function [19]. In addition, myomiRs have also been found to possess tumor suppressor function in over 14 human cancers [20], but have never been studied in a non-myogenic sarcoma. In this current study, we examined the role of miR-133a as a tumor suppressor in DDLPS.

## Methods

### Cell culture and reagents

Human LPS cell lines Lipo224B, Lipo246, Lipo863, and Lipo815 were established in our Sarcoma Research Laboratory as previously reported [21]. These cell lines were cultured in high glucose DMEM (Gibco, Carlsbad, CA, USA) with 10% fetal bovine serum (Atlanta Biologicals, Flowery Branch, GA, USA), supplemented with 0.1% Pen Strep (Gibco). Human white pre-adipocytes (HWP), derived from visceral adipose tissue, were obtained from PromoCell and cultured in mesenchymal stem cell medium, supplemented with the manufacturer’s supplemental mix. Poietics Preadipocytes (PreAdip) derived from visceral fat from normal human donors were obtained from Lonza (Walkersville, MD, USA) and cultured in manufacturer’s Preadipocyte Growth Media. Mature adipocytes were differentiated from preadipocytes by adding the manufacturer’s supplemental mix to Preadipocyte Growth Media according to manufacturer’s suggestions. All cell lines were cultured in normal conditions at 37 °C in a 5% CO_2_ humidified chamber.

### Transfection and lentiviral infection

For lentivirus production, lentiviral constructs were purchased from Systems Biosciences (Mountain View, CA, USA). Packaging of the lentiviral constructs was performed using the pPACKH1 Lentivector Packing KIT (Catalog No. LV500A-1) according to the manufacturer’s recommendation. DDLPS cells were transduced with either an empty lentivirus (Catalog No. CD511B-1) or a pre-miR-133a (Catalog No. PMIRH133a1PA-1). FACS-mediated cell sorting based on GFP expression was performed 72 h post-transduction, and miR-133a expression was evaluated by real-time PCR (Applied Biosystems, Foster City, CA, USA).

### RNA isolation and quantitative real-time PCR

RNA was isolated using a GeneJET RNA Purification Kit (Thermo Fisher Scientific, Waltham, MA, USA) and real-time PCR was performed using the StepOne Plus Detection System (Applied Biosystems). RNA concentration and purity were measured using the NanoDrop 2000 (Thermo Fisher Scientific). Real-time PCR for mature miRNA expression was performed using Taqman miRNA assays (assay ID: 002246, miR-133a; 001973, U6 snRNA) (Applied Biosystems). 50 ng total RNA was converted to first-strand cDNA with miRNA-specific primers, followed by real-time PCR with Taqman probes. All samples were normalized to U6 snRNA. Real-time PCR was also performed for mRNA expression. 1 μg of total RNA were used for synthesizing cDNA using iScript cDNA Synthesis Kit (Bio-Rad Laboratories, Hercules, CA, USA) in an iCycler (Bio-Rad Laboratories), and real-time PCR reactions were performed using SYBR Green (Roche, Indianapolis, IN, USA) with the following primer pairs: B2M: 5′-GAATTCACCCCCACTGAAAA-3′ (forward), 5′-CCTCCATGATGCTGCTTACA-3′ (reverse); CTGF: 5′-GAAGCTGACCTGGAAGAGAAC-3′ (forward), 5′-CGTCGGTACATACTCCACAG-3′ (reverse); MFN2: 5′-AGCTACACTGGCTCCAACTG-3′ (forward), 5′-GAGCTCACTGTCCAACCAAC-3′ (reverse). The ΔΔCt method was applied for all real-time PCR analysis.

### Proliferation and cell death assays

DDLPS cells were seeded in 96-well plates (TPP) at a density of 3000–5000 cells per wells and time-lapse live cell imaging was conducted in 37 °C and 5% CO_2_ conditions using the IncuCyte Zoom (Essen Bioscience, Ann Arbor, MI, USA) system, where 4 high-definition phase-contrast images were collected from each well every hour. IncuCyte Zoom 2015A software (Essen Bioscience) was used for analysis to apply a mask that determined the percent confluence based on the phase-contrast images. The percent confluence was normalized to the initial seeding confluence. For cell death assay, DDLPS cells were seeded as described above and stained with TOTO-3 (Molecular Probes, Eugene, OR, USA), a membrane impermanent nucleic acid probe. TOTO-3 fluorescence was measured by IncuCyte Zoom and the number of fluorescent cells was counted.

### Cell cycle assays

DDLPS cells were synchronized by contact inhibition and serum deprivation. Cells were grown to confluence and subsequently cultured with 0.1% fetal bovine serum in DMEM for 36 h. Cells were trypsinized and plated in 6-well plates at 2 × 10^6^ cells per well in normal 10% fetal bovine serum media. Cells were trypsinzed at time points of 0, 15, 24, 33, 36, and 48 h and subsequently stained using a Propidium Iodide Flow Cytometry Kit (Abcam, Cambridge, UK) according to manufacturer’s recommendation. DNA content analysis was performed by flow cytometry using Amnis FlowSight Imaging Flow Cytometer (End Millipore, Burlington, MA, USA) and IDEAS analysis software (End Millipore).

### Migration assays

DDLPS cells were plated in 96-well ImageLock plates (Essen Bioscience) at density of 1.6 × 10^4^ cells per well in the presence of mitomycin C (1 μg/mL, received from Dr. Dario Palmieri) to inhibit proliferation. After 12 h, Incucyte WoundMaker (Essen Bioscience) was used to make a uniform scratch in all 96 wells simultaneously. After rinsing with PBS, cells were placed in IncuCyte Zoom for time-lapse live cell imaging every hour for 24 h. Incucyte Scratch Wound Cell Migration Software Module (Catalog No. 9600-0012) was used to apply a mask based on phase-contrast that analyzed the percentage of the wound infiltrated by the cells and determined the relative wound density (normalizing for initial density).

### Xenograft

SCID hairless mice were purchased from Charles River Laboratories (Wilmington, MA, USA). Mice were housed under barrier conditions under IACUC protocol #2014A00000085. 1–2 × 10^6^ cells were injected subcutaneously into the flank and tumor size was measured using calipers twice per week.

### Metabolism assays

DDLPS cells were plated in XF24 cell culture microplates (Agilent, North Billerica, MA, USA) at densities of 2–4 × 10^4^ cells per well. After overnight incubation, cells were washed using XF base media (Agilent) supplemented with glucose (10 mM, Sigma, St. Louis, MO, USA), pyruvate (2 mM, Gibco), and l-glutamine (2 mM, Gibco). Cells were incubated for 1 h in a non-CO_2_ incubator. Cells were placed in Seahorse XFe24 Bioanalyzer (Agilent) for calibration and equilibration. MitoStress Test and GlycoStress Test were performed using glucose (10 mM, Sigma), oligomycin (1 μM, Sigma), FCCP (2 μM, Sigma), rotenone (2 μM, Sigma), and 2-deoxy-d-glucose (50 mM, Sigma).

For ex vivo metabolism studies, DDLPS tissues were obtained from subcutaneous xenograft tumor models as described above. After resection of DDLPS tumor, a biopsy punch (Integra LifeScience, Plainsboro, NJ, USA) was used to take a tissue core. The tissue core was embedded in 6% Low Melting Point Agarose (Invitrogen, Carlsbad, CA, USA) and sliced at 200 μM thickness using Vibratome 1000 Tissue Sectioning System (TPI, St. Louis, MO, USA). Tissue slices were placed in XF24 islet capture microplates with XF base media (Agilent) including 10 mM glucose, 2 mM pyruvate, and 2 mM glutamine and incubated for 1 h in a non-CO_2_ incubator. Seahorse XFe24 Bioanalyzer was used for measuring oxygen consumption rate.

### Statistical analysis

All quantitative data were presented as the mean ± SEM. GraphPad Prism 6 (GraphPad Software, Inc., La Jolla, CA, USA) was used for statistical analysis including two-tailed student’s *t* test, one-way ANOVA test, and two-way ANOVA test. p < 0.05 was considered to be statistically significant.

## Results

### miR-133a is underexpressed in DDLPS cell lines and liposarcoma tissues

**Fig. 1:**
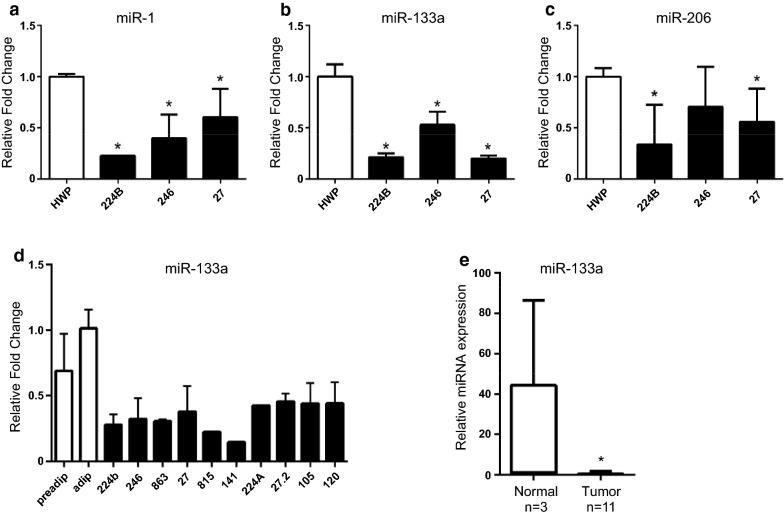
miR-133a is under expressed in DDLPS cell lines and liposarcoma tissues. **a**–**c** Expression levels of miR-1 (**a**), miR-133a (**b**), and miR-206 (**c**) were measured using real-time RT-PCR in human white preadipocyte cell line (HWP) and DDLPS cell lines (224B, 246, 27). Fold changes were calculated with the 2^−ΔΔCT^ method, using U6 snRNA as a housekeeping gene. Data are plotted as mean ± SEM for each miRNA performed in triplicate. *p < 0.05. **d** Real-time RT-PCR analyzed miR-133a expression level in a DDLPS cell line panel, along with preadipocytes (preadip) and adipocytes (adip) used as normal controls. Fold changes were calculated with the 2^−ΔΔCT^ method, using U6 snRNA as a housekeeping gene. Data are plotted as mean ± SEM. **e** Human tissues were analyzed by real-time RT-PCR for miR-133a expression. Tumor tissue included 11 liposarcomas and normal tissue included three normal adjacent tissues. Data are plotted as box and whisker plot. *p < 0.05

Previous results showed that miR-1, miR-133a, and miR-206 were under expressed in paired liposarcoma tumor tissues compared to their adjacent normal tissues [12]. Consistent with these results, we observed that miR-1, miR-133a, and miR-206 were also underexpressed in DDLPS cell lines 224B, 246, and 27 compared to control human preadipocyte cells (Fig. 1a–c). Interestingly, miR-133a, plays a role in adipocyte differentiation [14, 15], and amongst the other myomiRs was strongly downregulated in DDLPS cell lines compared to control cells. To expand our analysis, we focused our study on the expression of miR-133a in a larger cohort of DDLPS cells lines (n = 10). Similar to our original finding, miR-133a was significantly underexpressed in DDLPS cells compared to control adipose (Fig. 1d). Furthermore, miR-133a was significantly under expressed in unpaired human liposarcoma tumor tissues compared to normal fat (Fig. 1e). Such data suggest that miR-133a might function as a tumor suppressor in human liposarcoma.

### miR-133a overexpression is associated with decreased cell growth of DDLPS cells in vitro

**Fig. 2:**
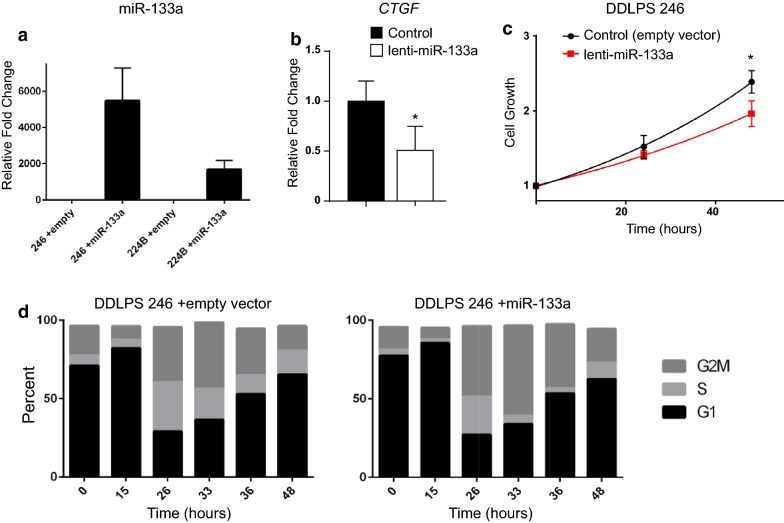
DDLPS cells reconstituted with miR-133a exhibit reduced cell growth in vitro. **a** Over expression of miR-133a validated using real time RT-PCR. Data are plotted as mean ± SEM for each miRNA performed in triplicate. *p < 0.05. **b** Real time quantitative PCR analysis was performed with primers against the *CTGF* gene. Primers to *B2M* were used to normalize the samples. Error bars represent standard error of mean for the replicate values. **c** Cell growth shown as measured by determining percent confluence from phase-contrast images normalized to plating density. DDLPS cell line 246 was transduced with lenti-miR-133a (red) or control (black). Two-way ANOVA was performed to determine statistical significance (p < 0.05). Data are shown as mean ± standard error of mean from three independent experiments. **d** Cell cycle progression analysis shown as distribution of cell phase at 15, 26, 33, 36, and 48 h time points. Data are representative of three independent experiments

To test the relevance of miR-133a expression in DDLPS, we stably expressed miR-133a using lentiviral pre-miR-133a transduction in human DDLPS cells (Fig. 2a). To validate the relevance of miR-133a overexpression, we probed for a previously identified target of this miRNA called connective tissue growth factor (*CTGF*) [22]. As anticipated, miR-133a overexpression reduced the levels of *CTGF* in DDLPS cells compared to control vector cells (Fig. 2b). Compared to empty vector control, cell growth was significantly reduced (p < 0.05) in miR-133a-overexpressing DDLPS cell lines (Fig. 2c). We subsequently inquired whether changes in cell growth were mediated by a defect in the cell cycle. Using the same DDLPS cell line stably transduced with miR-133a or control vector, we performed propidium iodide staining to study the kinetics of cell progression via the cell cycle. Results showed that DDLPS cells expressing miR-133a were delayed in entering S phase at 33 and 36 h (Fig. 2d).

We next investigated whether expression of miR-133a impacted cell death. By TOTO-3 staining for nucleic acid, we were unable to observe a difference in cell death between vector control and miR-133a expressing DDLPS cells (data not shown). We also investigated the potential role of miR-133a in promoting DDLPS cell migration. However, results from a wound scratch assay did not show significant differences between vector and miR-133a expressing DDLPS cells (Additional file 1: Figure S1a, b). Together, these results suggested that miR-133a is capable of impairing DDLPS cell growth, but this regulation correlated only modestly with the cell cycle, but not with cell death or cell migration.

### DDLPS cells re-expressed with miR-133a showed increased mitochondrial function and decreased glycolytic capacity

**Fig. 3:**
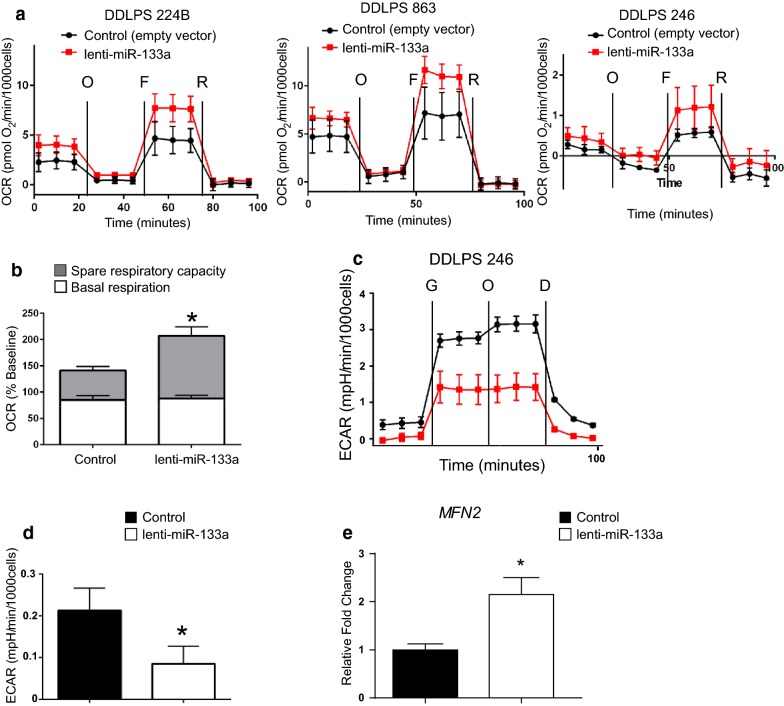
DDLPS cells reconstituted with miR-133a show shifted mitochondrial function linked to energy balance. **a** Mitostress Test was performed in DDLPS cell lines 224B, 863, and 246. **b** OCR values shown after injection of FCCP relative to baseline OCR measurement (n = 3). *p < 0.05. **c** Glycostress Test was performed in DDLPS cell line 246. **d** Basal glycolysis was measured by baseline ECAR subtracted by non-glucose-derived ECAR (ECAR values after injection of 2-deoxyglucose). **e** Real time quantitative PCR analysis was performed with primers against the *MFN2* gene. Primers to *B2M* were used to normalize the samples. Error bars represent standard error of mean for the replicate values. *D* 2-deoxy-d-glucose, *ECAR* extracellular acidification rate, *F* carbonyl cyanide-*4*-(trifluoromethoxy)phenylhydrazone), *G* glucose, *OCR* oxygen consumption rate, *O* oligomycin, *R* rotenone

Having observed a modest effect on proliferation, but no effect on cell death or migration, we investigated whether miR-133 expression in DDLPS affected the metabolic profile of these cells. Using the Seahorse bioanalyzer, Mito Stress tests revealed that reconstitution of miR-133a in DDLPS cells led to higher maximal respiration from an increased oxidative phosphorylation (OXPHOS) capacity (Fig. 3a, b). Correspondingly, a Glyco Stress test showed that expression of miR-133a decreased glycolysis in DDLPS, compared to vector control cells (Fig. 3c, d). Since high mitochondrial respiratory capacity is associated with mitochondrial fusion [23], we examined the expression of mitofusin 2 (*MFN2*), a gene which is essential for mitochondrial fusion and also regulates glucose homeostasis [24]. We found increased expression of *MFN2* associated with reconstitution of miR-133a in DDLPS cells (Fig. 3e). Together, these results indicate that re-constitution of miR-133a in DDLPS cells promotes a reduction in glycolysis and an increase in OXPHOS.

### Reconstitution of miR-133a does not reduce DDLPS tumor growth but promotes a metabolic shift in vivo

**Fig. 4:**
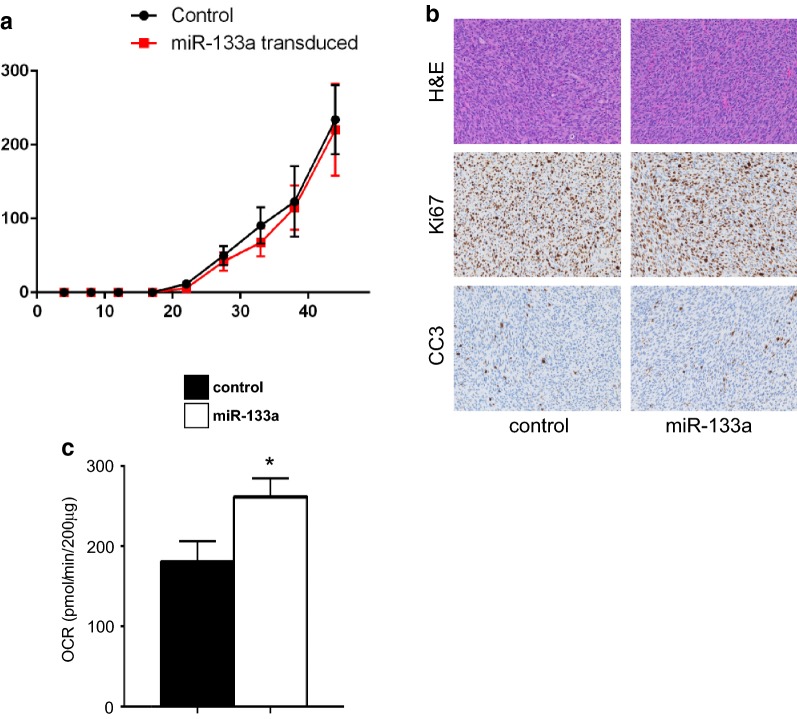
Re-expression of miR-133a impacts DDLPS tumor cell metabolism. **a** DDLPS 246 cells were subcutaneously injected into SCID mice (n = 6 per condition). Tumor area was measured twice per week using calipers. **b** H&E staining along with immunohistochemistry staining for Ki67 and cleaved caspase 3 (CC3). **c** OCR measurements of ex vivo tumor tissues shown normalized to dried tissue mass

To examine the in vivo relevance of our findings, we subcutaneously injected human DDLPS cells stably expressing miR-133a or a scrambled miRNA control in SCID mice [10, 25, 26]. Reconstitution of miR-133a in DDLPS cells did not affect tumor growth (Fig. 4a) nor was there a difference in Ki67 by immunohistochemistry staining. This suggested that miR-133a does not impact DDLPS cellular proliferation in vivo (Fig. 4b). In addition, consistent with in vitro findings, miR-133a showed no difference in cleaved caspase 3 (CC3) by immunohistochemistry staining versus control DDLPS cells (Fig. 4b), which confirmed that loss of miR-133a does not mediate tumor cell growth or survival.

To determine whether miR-133a has an impact on tumor metabolism as we had observed in culture conditions, we profiled dissected DDLPS tumors from mice at endpoint and analyzed fresh, unfixed, tumor sections directly on the Seahorse analyzer. Significantly, reconstitution of miR-133a increased the baseline oxygen consumption rate in DDLPS tumors (Fig. 4c), directly reflecting an increase in OXPHOS. These data support the concept that miR-133a functions as a regulator of oxidative metabolism in adipocyte cells and loss of this miR leads to a shift in metabolism favoring a Warburg effect in DDLPS tumors.

## Discussion

This study was performed to determine the role of miR-133a in DDLPS. Our findings support the contention that miR-133a is decreased in DDLPS compared to normal cells. By stably reconstituting miR-133a in human DDLPS cells, we observed a decrease in proliferation and a shift in metabolism from a glycolytic to an OXPHOS phenotype. When DDLPS cells expressing miR-133a were injected in a murine in vivo xenograft model, tumor growth was unchanged, but a shift towards oxidative metabolism was retained. This implied that in DDLPS, loss of miR-133a contributes to the shift in tumor cells utilizing glycolysis as a main energy source. The tumor suppressor role of miR-133a and its family (including miR-1, miR-133b, and miR-206), were originally described in the muscle relevant cancer, rhabdomyosarcoma, due to their multiple roles in promoting skeletal muscle differentiation. Following their discovery in rhabdomyosarcoma, other tumor types were found to be associated with loss of these miRs [20]. With miR-133a, this included bladder, breast, colorectal, and gastric cancers [26–31]. In breast cancer, loss of miR-133a promotes cell cycle and cell proliferation due to the increase in the epithelial growth factor receptor (EGFR), identified as a miR-133a target [27]. In colorectal cancer, miR-133a represses tumor growth by targeting LASP1 [29] and RFFL [30], which function by inducing cell proliferation and inhibiting the p53/p21 signaling pathway, respectively.

In contrast, in DDLPS, we did not find miR-133a to be strongly involved in cell cycle regulation, nor were any migration or cell survival effects observed. Instead miR-133a seemed to play a more vital role in regulating metabolism. This is consistent with miR-133a function to promote glucose transport in cardiac cells by targeting KLF15 [32], which acts as a negative regulator of the glucose transporter, GLUT4, and to induce the differentiation of brown fat by targeting Prdm16 in skeletal muscle progenitor cells [16].

Metabolic reprogramming has emerged as one of the key hallmarks of cancer [30, 31]. A distinctive feature of tumor cells compared to normal cells is their ability to thrive in a variety of microenvironments including hypoxic and nutrient-depleted backgrounds. The most fundamental metabolic alteration is that cancer cells exhibit aerobic glycolysis, a process in which cells increase glucose consumption and undergo glycolysis even in the presence of oxygen [33, 34]. Although the ATP yield per glucose molecule is much lower through glycolysis than through aerobic respiration, cancer cells can utilize this pathway to expediently acquire energy to support rapid cellular proliferation [35]. This glycolytic phenotype was proposed by Otto Warburg to be associated with weak mitochondrial oxidative phosphorylation [36], but this view has been challenged by investigations showing that mitochondrial oxidative phosphorylation is intact in most cancers [37]. The increased dependence of cancer cells on aerobic glycolysis led to development of therapeutic strategies that pharmacologically inhibit glycolysis [33]. However, the potential therapeutic benefits will likely be context- and tumor type-dependent based upon whether the cancer cells are dependent on glycolysis or mitochondrial oxidative phosphorylation [38, 39].

An interesting result in our study was that miR-133a impacted metabolism of tumor cells in vivo. To the best of our knowledge, our findings are the first to show results from live tumor tissue utilizing the Seahorse Bioanalyzer. We adopted this method from a previous study where we successfully measured the oxygen consumption rate of intact skeletal muscle [40]. We found that overexpressing miR-133a in DDLPS cells increased OXPHOS both in vitro and ex vivo. However, this shift in metabolism was not sufficient to alter tumor growth. It is possible that the regulation on metabolism by miR-133a might have stronger implications earlier in the pathogenesis of DDLPS. Support for this notion comes from a genetically engineered mouse model of DDLPS, where Notch receptor activation in adipocytes induced DDLPS [41]. In this DDLPS model, Notch activation suppressed lipid metabolism pathways, and administration of the synthetic PPARγ ligand rosiglitazone reversed the metabolic alteration and dedifferentiation in DDLPS tumors. It is therefore possible that increased expression of miR-133a could comparably alter lipid metabolism pathways and thereby prevent early progression of DDLPS.

## Conclusion

In this study, we demonstrated that overexpression of miR-133a in DDLPS induces a shift in metabolism from a glycolytic to an oxidative phenotype. We have further shown in an in vivo xenograft model that a shift to oxidative metabolism is retained. These findings suggest the possibility that miR-133a could function as a regulator of oxidative metabolism in DDLPS tumors.

## Additional file

**Additional file 1: Figure S1.** DDLPS cells reconstituted with miR-133a do not show differences in migration. **a** Scratch wound assay was performed in DDLPS cell line 246 transduced with lenti-miR-133a (top) or control (bottom). The initial scratch wound mask was created immediately after wound creation and is shown in blue. Phase contrast-images were taken as cells (yellow) migrate in to the wound region over time. **b** Relative wound density was calculated by Incucyte Scratch Wound Cell Migration Software.

## Abbreviations

DDLPS: dedifferentiated liposarcoma
EGFR: epidermal growth factor receptor
HWP: human white preadipocyte
OXPHOS: oxidative phosphorylation
WDLPS: well-differentiated liposarcoma
