# Supplementary figures and images for: miR-133a function in the pathogenesis of dedifferentiated liposarcoma
Source: Cancer Cell Int. 2018 Jun 26;18:89. doi: 10.1186/s12935-018-0583-2 (PMC6019219; doi:10.1186/s12935-018-0583-2)

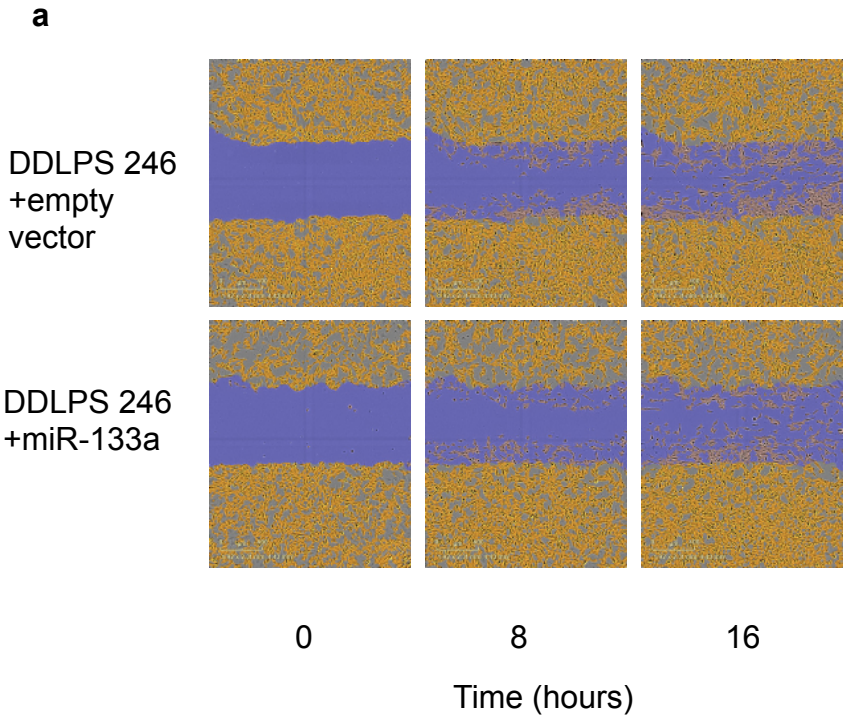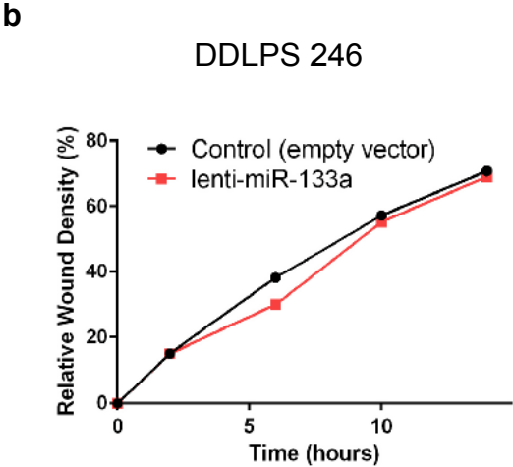

Supplement: Supplementary file 1 — Additional file 1: Figure S1. DDLPS cells reconstituted with miR-133a do not show differences in migration. a Scratch wound assay was performed in DDLPS cell line 246 transduced with lenti-miR-133a (top) or control (bottom). The initial scratch wound mask was created immediately after wound creation and is shown in blue. Phase contrast-images were taken as cells (yellow) migrate in to the wound region over time. b Relative wound density was calculated by Incucyte Scratch Wound Cell Migration Software. [file 12935_2018_583_MOESM1_ESM.pdf]
